# The value of ^18^F-FDG PET/CT in the systemic evaluation of patients with Rosai–Dorfman disease: a retrospective study and literature review

**DOI:** 10.1186/s13023-023-02711-8

**Published:** 2023-05-13

**Authors:** Xinyu Lu, Rongxi Wang, Zhaohui Zhu

**Affiliations:** 1 Department of Nuclear Medicine, Beijing Key Laboratory of Molecular Targeted Diagnosis and Therapy in Nuclear Medicine, Peking Union Medical College Hospital, Chinese Academy of Medical Sciences and Peking Union Medical College, Beijing, 100730 China; 2 State Key Laboratory of Complex Severe and Rare Diseases, Beijing, 100730 China

**Keywords:** Rosai–Dorfman disease, Lesion identification, ^18^F-FDG PET/CT, Non-Langerhans cell histiocytosis

## Abstract

**Background:**

Rosai–Dorfman disease (RDD) is a rare form of non-Langerhans cell histiocytic disease. The aim of this study was to review the characteristics of RDD using ^18^F-FDG PET/CT and determine its efficacy in the disease management.

**Results:**

A total of 28 RDD patients underwent 33 ^18^F-FDG PET/CT scans for systematic assessment and follow-up. The common involved sites included the lymph nodes (17, 60.7%), upper respiratory tract (11, 39.3%), and skin (9, 32.1%). Five patients had more lesions detected in PET/CT images than in CT and/or MRI, including inapparent nodules (n = 5) and bone destruction (n = 3). After thorough treatment evaluation using PET/CT, the treatment strategies of 14 patients (14/16, 87.5%) were changed. Five patients underwent PET/CT twice during follow-up and the SUVs were significantly decreased (15.3 ± 3.4 vs. 4.4 ± 1.0, p = 0.02), which demonstrated disease improvement.

**Conclusions:**

^18^F-FDG PET/CT contributed to displaying the holistic characteristics of RDD, in particular during initial assessment, treatment strategy adjustment, or efficacy evaluation, and could compensate for some disadvantages of CT and MRI images.

## Background

Rosai–Dorfman disease (RDD) is a rare form of histiocytic disease that was first reported in 1965 [1]. It was subsequently characterized by Rosai and Dorfman in 1969 [2] and finally classified as non-Langerhans cell histiocytosis. This heterogeneous syndrome has different clinical phenotypes that incorporates sporadic, familial, and cutaneous groups [3]. According to the clinical features, sporadic RDD can be further classified into classic nodal-involved, extranodal, immune disease-associated, and neoplasia-associated RDD.

As an orphan disease, the prevalence of RDD is approximately 1:200,000 [4] with a higher morbidity rate in male African patients [5]. Its cutaneous form is reportedly common in female Asian patients [6]. The classic type always presents with massive, and painless systemic lymph nodes, weight loss, fever, and night sweats [7, 8]. Extranodal RDD has been reported in 43% of cases and usually occurs in older adult patients with nodal involvement [9]. Extranodal affected sites include the skin, central nervous system (CNS), orbital tissues, nasal cavity, paranasal sinuses, intrathoracic lesions, and bones [10]. A defining pathological feature of RDD is sinusoidal expansion with diffuse infiltration of histiocytes in enlarged nodes. Emperipolesis, namely intact leukocytes in histiocyte cytoplasm, is regarded as a helpful but non-specific finding [11]. Extranodal lesions are similar to nodal RDD, but represent with more fibrosis, sclerosis, fewer histiocytes, and more subtle emperipolesis. Specific immunohistochemical markers, such as S100, CD68, and CD1a, can distinguish RDD from Erdheim–Chester disease (ECD). Additionally, appropriate clinical and radiological contexts are essential, especially in cases with comorbidities in which histology may be atypical and confusing [12].

In addition to the significance of differential diagnosis, imaging examinations in patients with RDD are important for biopsy-site selection and evaluation of disease severity. Conventional imaging examinations, such as magnetic resonance imaging (MRI), ultrasound and computed tomography (CT), are recommended depending to the symptoms or organ involvement of the patients. ^18^F-fluorodeoxyglucose (FDG) positron emission tomography/computed tomography (PET/CT) may be beneficial for initial staging and treatment assessment in patients with RDD. However, a consensus on the ideal disease assessment has not yet been reached. Here, ^18^F-FDG PET/CT findings of RDD patients were reviewed and compared with conventional imaging results from the corresponding period. In addition, a literature review is presented, specifically on the efficacy of PET/CT in RDD management.

## Materials and methods

### Study design and patients

Medical records from January 2012 to November 2021 were reviewed to identify patients with RDD. The inclusion criteria were: (1) histopathological identification of RDD; and (2) at least one ^18^F-FDG PET/CT scan performed. Initially, 175 patients with a suspected diagnosis of RDD were included. After review of the histopathological results, 162 patients were diagnosed with RDD with certainty. In total, twenty-eight patients met all criteria, while most of the others had only cutaneous lesions without any further imaging examinations. After a period of treatment, five patients had a second PET/CT scan. Demographic data, physical examination, histopathological features, and treatment information were collected.

### ^18^F-FDG PET/CT imaging

Patients all fasted for 4–6 h before the procedure and their blood glucose levels were below 200 mg/ml. Then they were injected with ^18^F-FDG with a dosage of 5.2–7.4 × 10^6^ Bq/kg and rested for 60–90 min. ^18^F-FDG PET/CT scans were performed using a combined PET/CT biograph (Siemens, Germany), with patients in the supine position, from the skull vertex or base to the mid-thigh level (1.5–2 min/bed position, 5–6 bed positions). Low-dose CT scans were obtained at the beginning for attenuation correction and anatomical reference (120 keV, 50 mAs; 3 mm slice thickness).

### Image analysis

All PET/CT images were reviewed by two nuclear medicine physicians, and a consensus was reached in cases of disagreement. The SUV was measured by selecting a region of interest that showed non-physiological hypermetabolism greater than the background. If available, CT and/or MRI performed within a time window of one month were compared with ^18^F-FDG PET/CT.

### Literature review

The literature review complied with the rule of Preferred Reporting Items for Systematic Reviews and Meta-Analyses (PRISMA). Studies that fulfilled the following criteria were included: (1) prospective or retrospective reports on the utility of ^18^F-FDG PET/CT in RDD published online in English, (2) including at least four participants and a detailed description of the ^18^F-FDG PET/CT results.

**Fig. 1:**
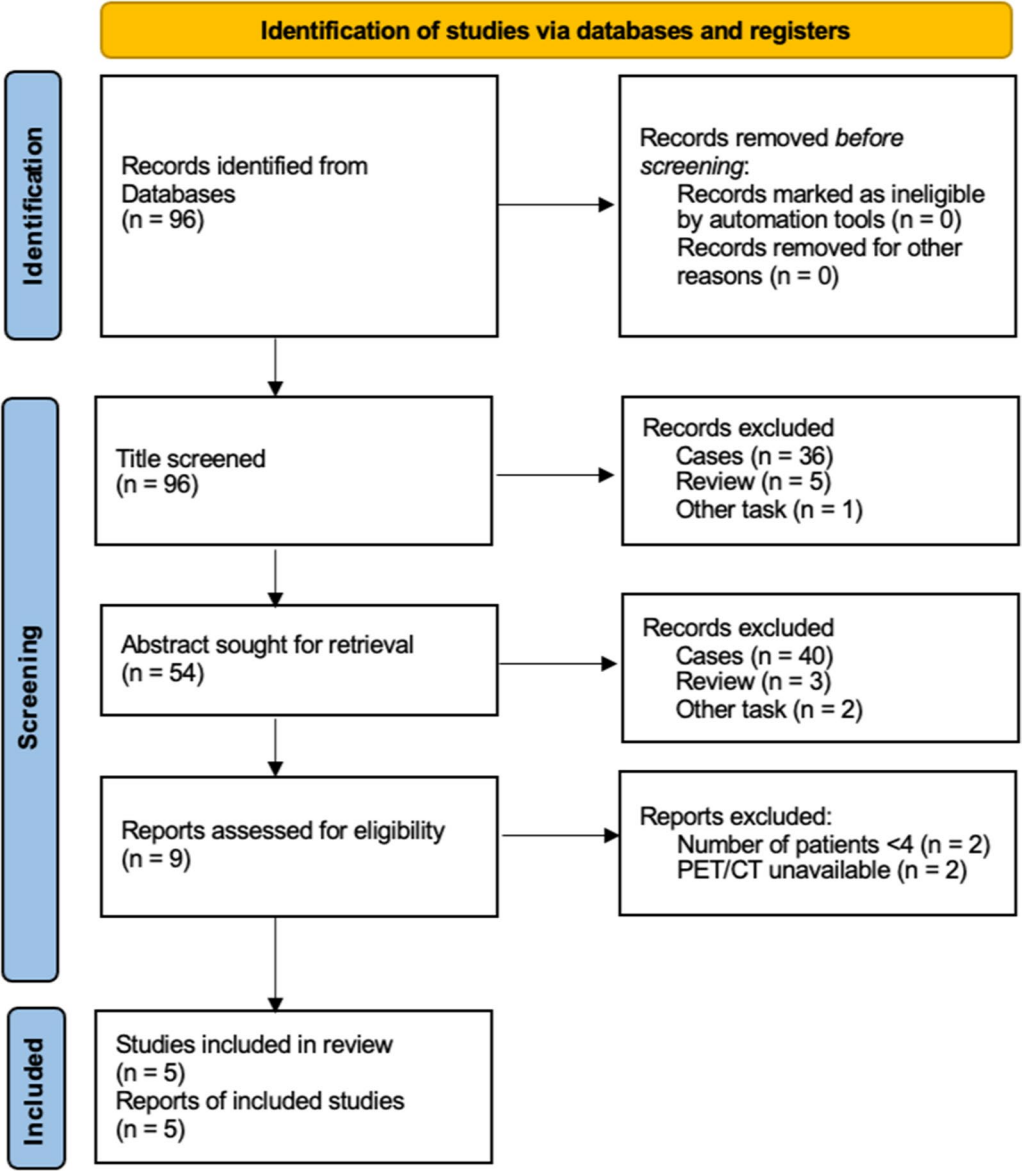
Study selection flowchart for literature review

The literature search was limited to the period from January 1, 2000, to November 1, 2022. The PubMed database were searched using a combination of the following keywords: (1) positron emission tomography or PET and (2) Rosai–Dorfman disease or Sinus Histiocytosis with Massive Lymphadenopathy. In total 96 studies were retrieved during the main search, and all abstracts were screened for further selection. Five studies were finally included based on the review criteria (Fig. 1). The following information was extracted from these studies: number of patients, sex, age, information on different imaging modalities, diagnostic criteria, treatment, and follow-up.

### Statistical analysis

Data were presented as mean and standard deviation (SD) for continuous variables, while categorical variables were presented as number and proportion (%). The change of SUVmax during follow-up was analyzed using a paired t-test. The SUVmax of groups with different disease features were compared using t-tests or Mann–Whitney U tests. The proportions of involved sites of different cohorts were compared using chi square tests or Fisher’s tests. Statistical significance was set at a p value < 0.05. Data analyses were conducted using SPSS software version 23 (IBM Corp, Armonk, NY, USA).

## Results

### Characteristics of patients and PET/CT

A total of 28 patients (18 men and 10 women) with an average age of 40.1 ± 21.4 years and available PET/CT results were included (Table 1). According to the scan results, two patients had only nodal lesions, 11 patients had only extranodal lesions, and 15 patients had both nodal and extranodal involvement. The lymph nodes (17, 60.7%) were the most common lesion locations (Table 2). The most commonly involved extranodal regions were the upper respiratory tract (11, 39.3%), skin (9, 32.1%), bone (6, 21.4%), and CNS (5, 17.9%).

**Table 1:** ^18^F-FDG PET/CT findings and treatment of patients

Case	Age/gender	Disease condition	Pre-PET treatment	Involved organs/tissues detected by PET/CT	Post-PET treatment
1	56/M	Suspected recurrence	Resection	Paranasal sinus, nasopharynx and lymph node	Thalidomide and glucocorticoid
2	33/M	Undiagnosed	–	Multiple intracranial lesions and lymph nodes	Resection, lenalidomide and glucocorticoid
3	70/M	Suspected recurrence	Resection	Paranasal sinus, bilateral nasal cavity and laryngeal	Thalidomide
4	28/F	Suspected recurrence	Biopsy, glucocorticoid	Bilateral cervical lymph nodes	Resection
5	35/M	Newly diagnosed	Resection, glucocorticoid	Orbital apex and nasopharyngeal	Lenalidomide and glucocorticoid
6	26/F	Suspected recurrence	Resection	Nasopharyngeal cavity, superior turbinate, subglottic nodule, subcutaneous nodules and lymph nodes	Lenalidomide and glucocorticoid
7	11/M	Newly diagnosed	Biopsy	Pituitary, nasal cavity, nasopharyngeal and multiple bone lesions	Glucocorticoid
8	22/M	Undiagnosed	–	Throat and lymph node	Resection and thalidomide
9	57/M	Newly diagnosed	Resection	Nasal cavity, subglottic nodule and pituitary	Thalidomide
10	48/F	Undiagnosed	–	Pulmonary trunk lumen, ascending aortic root wall and lymph nodes	Resection
11	38/M	Newly diagnosed	Biopsy	Subcutaneous nodule	Resection
12	27/F	Newly diagnosed	Resection	Lymph nodes	Observation
13	21/F	Newly diagnosed	Resection	Nasal cavity, paranasal sinus, subcutaneous nodules and lymph nodes	Lenalidomide and glucocorticoid
14	42/M	Newly diagnosed	Resection	Lacrimal gland, parotid gland, nasopharynx, pleura, pericardium, bone, subcutaneous nodules and lymph nodes	Lenalidomide and glucocorticoid
15	37/M	Suspected recurrence	Biopsy, glucocorticoid	Multiple intracranial lesions	Cytarabine
16	32/F	Suspected recurrence	Glucocorticoid	Parotid gland	Lenalidomide and glucocorticoid
17	22/F	Newly diagnosed	Resection	No lesion	Observation
18	51/F	Undiagnosed	–	Multiple bone lesions and lymph nodes	Lenalidomide and glucocorticoid
19	70/M	Suspected recurrence	Resection, glucocorticoid, radiotherapy, immunosuppressor	Muscles, orbit, paranasal sinus, eyelids, plica vocalis and lymph nodes	Cytarabine
20	33/F	Newly diagnosed	Biopsy	Nasal cavity, trachea, adrenal nodule, subcutaneous nodules and lymph nodes	Glucocorticoid
21	53/M	Newly diagnosed	Biopsy	Multiple bone lesions, subcutaneous nodules and lymph nodes	Lenalidomide and glucocorticoid
22	65/M	Undiagnosed	–	Multiple intracranial lesions, high-density tissue in T3-4 spinal canal, subcutaneous nodule and lymph nodes	Cytarabine
23	63/F	Newly diagnosed	Resection, glucocorticoid, immunosuppressor	Muscles, bone lesion and subcutaneous nodules	Lenalidomide and glucocorticoid
24	53/M	Suspected recurrence	Resection, glucocorticoid	Extraocular muscles, eyelids, lacrimal glands, parotid gland, laryngeal cavity and perirenal soft tissue	Lenalidomide and glucocorticoid
25	60/M	Undiagnosed	–	Soft tissue in right atrium	Resection
26	14/M	Suspected recurrence	Biopsy, glucocorticoid, intravenous immunoglobulin	Lymph nodes and bone marrow	Glucocorticoid and methotrexate
27	18/M	Undiagnosed	–	Multiple intracranial lesions and lymph nodes	Cytarabine
28	39/M	Undiagnosed	–	Multiple bone lesions and lymph nodes	Lenalidomide and glucocorticoid

**Table 2:** Characteristics and comparison of our patients with those from the literature

Variables	Retrospective study (n = 28)	Literature review (n = 61)	p value
Age (years), mean (SD)	40.1 (17.4)	43.1 (21.4)	0.555
Male/female, n (%)	18/10 (64.3/35.7)	22/17 (56.4/43.6)	0.616
Disease distribution			
Lymph node, n (%)	17 (60.7)	29 (47.5)	0.248
Upper respiratory tract, n (%)	11 (39.3)	11 (18.0)	**0.038**
Skin, n (%)	9 (32.1)	14 (23.0)	0.358
Bone, n (%)	6 (21.4)	19 (31.1)	0.344
Central nervous system, n (%)	5 (17.9)	8 (13.1)	0.537
Paranasal sinus, n (%)	4 (14.3)	8 (13.1)	> 0.999
Eye, n (%)	4 (14.3)	3 (4.9)	0.200
Cardiovascular system, n (%)	2 (7.1)	0 (0)	0.097
Lung, n (%)	1 (3.6)	5 (8.2)	0.124
Treatment given before PET/CT, n (%)	16 (57.1)	–	–

Bold font indicates statistical significance

**Fig. 2:**
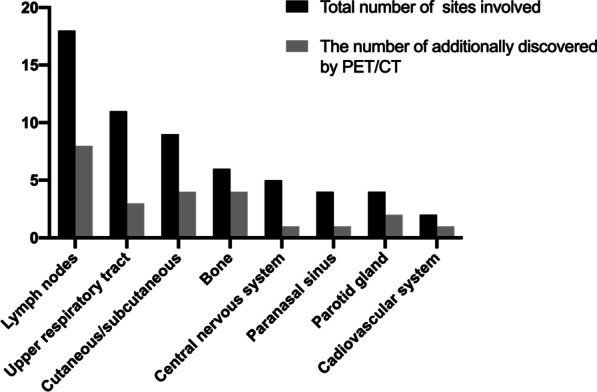
Involved organs and the lesions additionally detected by ^18^F-FDG PET/CT. Black bars: total number of cases with the sites involved, determined by a combination of imaging and clinical manifestations. Gray bars: the number of involvements additionally detected by ^18^F-FDG PET/CT

**Fig. 3:**
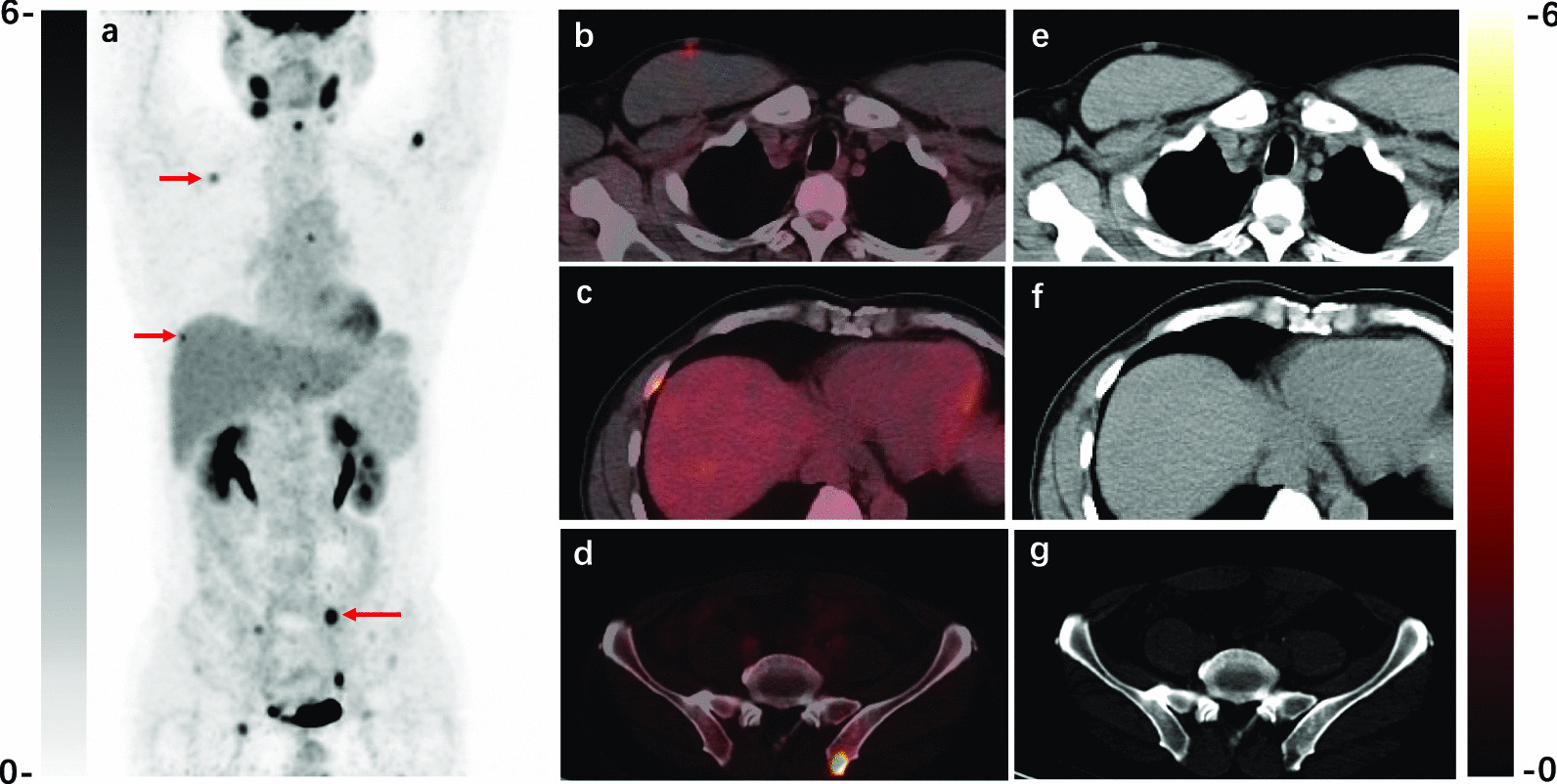
A typical RDD case with multisystem lesions revealed by ^18^F-FDG PET/CT. A 53-year-old man presenting with bilateral cervical lymph nodes enlargement was newly diagnosed with RDD by biopsy. The PET/CT images before treatment displayed hypermetabolic lymph nodes, subcutaneous nodules (**b, e**), and osteolytic lesions (**c, d, f, g**). He underwent chest CT before PET/CT, but these hypermetabolic nodules and bone lesions were neglected. The patient received lenalidomide and glucocorticoid due to multiple extranodal lesions

In 16 (57.1%) patients, newly involved sites that were not suggested by clinical symptoms were discovered after PET/CT scans. Previously undiscovered sites, including the lymph nodes and bones, are illustrated in Fig. 2. Twenty-four patients underwent CT and/or MRI scans within one month of their PET/CT scan. Five patients had additional lesions detected by PET, including inapparent nodules (n = 5) and bone destruction (n = 3). Figure 3 shows a typical case of previously hidden lesions revealed on PET/CT.

The SUVmax of all lesions was 12.1 ± 8.4. The specific SUV ranges of the involved sites are shown in Table 3. Among these lesions, the CNS, upper respiratory tract, paranasal sinus, and bone seemed to be more hypermetabolic with a mean SUVmax of over 10. The SUVmax of patients who underwent previous treatment was similar to that of patients who were untreated or underwent surgery only (11.8 ± 6.1 vs. 12.6 ± 11.9, p = 0.495), which is consistent with the fact that most patients relapsed or did not improve after taking medication at the time of the visit.

**Table 3:** SUV of different lesions on ^18^F-FDG PET/CT scans

Involved area	SUV range	SUV mean
Central nervous system	4.7–40.73	18.0
Eye	6–14.4	11.9
Upper respiratory	4.3–11.5	7.8
Paranasal sinus	9.3–20	14.2
Bone	4.4–21.4	11.0
Nodal	2.8–18	8.6
Cutaneous/subcutaneous	2.3–15.1	7.8

### Systemic assessment and treatment

A total of 19 (67.9%) patients underwent PET/CT before specific treatment for RDD. Before the histopathological diagnosis, eight cases underwent PET/CT scans for differential diagnosis and tissue biopsy in the hypermetabolism lesions. Based on PET/CT results, two patients with a single extranodal lesion only underwent surgical resection. Six patients with multiple extranodal lesions received chemotherapy (lenalidomide and glucocorticoid = 4, cytarabine = 2).

PET/CT was performed for whole body assessment in the other 11 patients (11/28, 39.3%) who were newly diagnosed with RDD. Besides 4 patients who had only undergone needle biopsy, seven patients had undergone surgical resection, and 2 of them had taken glucocorticoids with little effect. According to the PET/CT scans, three patients had only a single extranodal lesion and received no further treatment except resection. Eight patients, in whom multiple extranodal lesions were found, underwent medicine treatment (lenalidomide = 6, thalidomide = 1, and glucocorticoid = 1). In summary, patients with only lymph node involvement were treated with observation, those with a single extranodal lesion received surgical resection, and those with multiple extranodal lesions received chemotherapy.

Nine (32.1%) patients were suspected of disease recurrence or progression and had received treatment before. Eight patients showed recurrent FDG-avid lesions and four of them were confirmed by tissue biopsy. One patient with only nodal involvement continued glucocorticoids therapy while the other seven were switched to chemotherapy. Another patient was treated with methotrexate for combined systemic lupus erythematosus, without evident progression of only nodal-involved RDD. To sum up, the treatment of 14 of the 16 patients (87.5%) who had received treatment before was changed after a thorough assessment using PET/CT.

### Follow-up evaluation

**Fig. 4:**
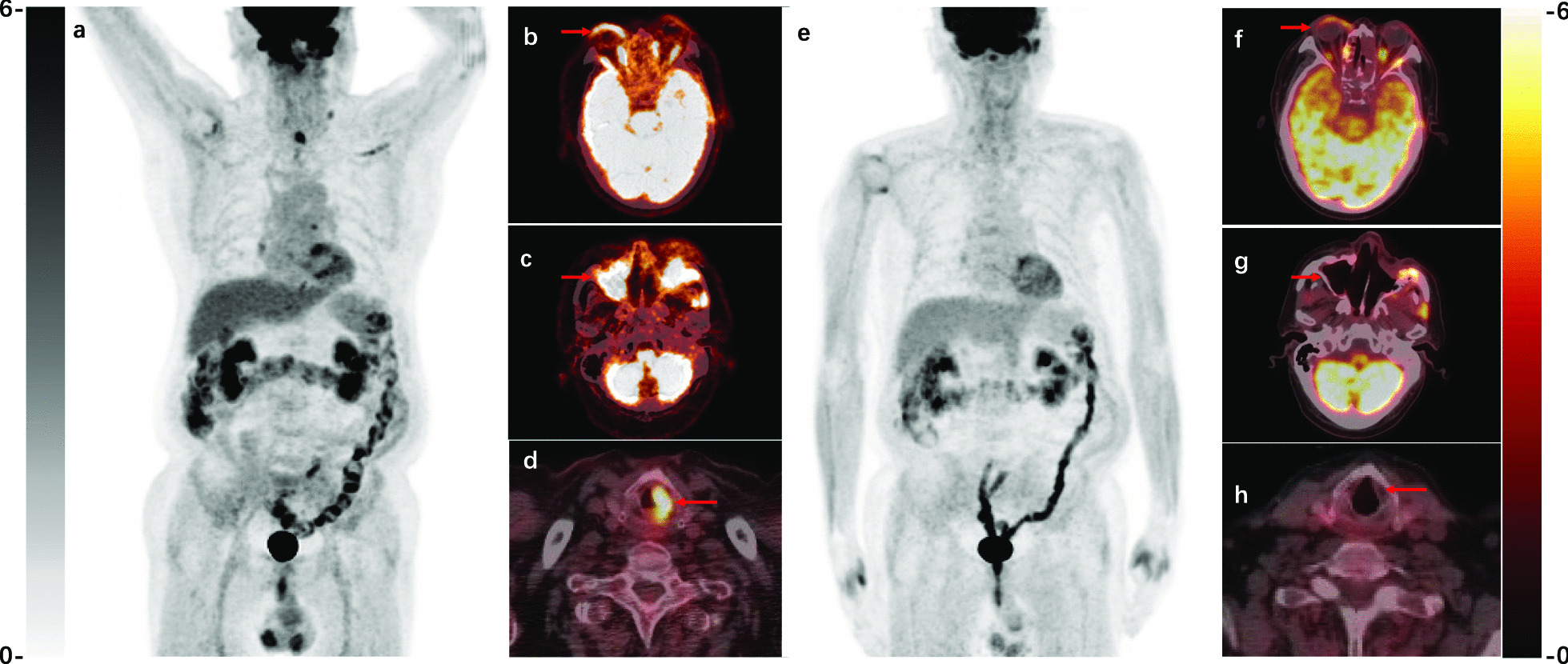
A typical case showing the utility of ^18^F-FDG PET/CT to follow-up of RDD. ^18^F-FDG PET/CT images of RDD with partial response in a 70-year-old man, after 4-month cytarabine. **a–d** Pretreatment PET/CT images displaying hypermetabolic eyelids (SUVmax 7.4), maxillary sinus (SUVmax 9.3) and left plica vocalis (SUVmax 9.0). **e–h** Follow-up scans showing lesion resolution with metabolic uptake and volume decrease: eyelids (SUVmax 4.3), maxillary sinus (SUVmax 5.7), left plica vocalis lesions disappeared

Five patients underwent a second PET/CT scan to evaluate the therapeutic response during follow-up. The mean time between the two scans was 9 ± 1.5 months. In all five patients, the lesion size and SUV decreased. An exemplary patient is shown in Fig. 4. There was a significant decrease of SUV between the two examinations (15.3 ± 3.4 vs. 4.4 ± 1.0, p = 0.02), which indicated disease improvement. As a result, the treatment of one patient was downgraded from cytarabine to lenalidomide.

No patient underwent CT again during follow-up. Nonetheless, six patients underwent a second enhanced MRI for evaluation of head lesions, including the brain (n = 2), eyes (n = 2) and parotid glands (n = 2). The time until this second MRI was 6.7 ± 3.8 months. Intensive lesions shrunk or decreased in all patients except for one who had parotid gland lesions that did not decrease significantly. In view of the high background values of normal brain tissue in ^18^F-FDG PET/CT, enhanced MRI can be used as a complement in such lesions.

### Literature review

After screening, five retrospective studies were included [13–17] that reported ^18^F-FDG PET/CT results of RDD (Table 4). There were 61 cases in which specific RDD lesions were observed on PET/CT. The most commonly involved organs were the lymph nodes (29, 47.5%), bone (19, 31.1%) and cutaneous/subcutaneous areas (14, 23.0%) (Table 2). The proportion of upper respiratory involvement was significantly higher in our center than in the literature (39.3% vs. 18.0%, p = 0.038).

**Table 4:** Characteristics of selected studies

Authors	Country	Study type	Patients with available PET/CT results	Mean age (years)	Male/female
Elshikh et al. [13]	USA	Retrospective	14	48.8	5/9
Vaidya et al. [14]	India	Retrospective	12	33.5	11/1
Raslan et al. [15]	Egypt	Retrospective	4	31.2	2/2
Mahajan et al. [16]	USA	Retrospective	22	NA*	NA
Sathyanarayanan et al. [17]	USA	Retrospective	9	52.1	4/5

*NA: not available

Compared with conventional imaging examination, some ^18^F-FDG non-avid lesions were found in six cases (Table 5), including breast/subcutaneous nodules, bone, lacrimal gland, and CNS lesions. Because of the lack of pathological properties, certain causes of these non-avid lesions, which could be comorbidities or outside the PET/CT field of view, remain unknown.

**Table 5:** Characteristics of FDG non-avid lesions

Source	Age (years)	Gender	^18^F-FDG PET/CT finding	Control imaging examination finding	Non-avid lesions
Elshikh et al. [13]	64	F	Non-avid left breast mass and non-avid subcutaneous mass in the left thigh	CT: ill-defined, isodense left breast mass	Left breast mass and subcutaneous mass in the left thigh
	50	F	Left parotid nodule with mild metabolic hyperactivity	Mammography: isodense nodule in the left breast	Left breast mass
	58	F	Lymphadenopathy and non-avid left pubic ramus lesion	CECT: axillary, diaphragmatic, retroperitoneal, mesenteric, portal lymphadenopathy, and lytic lesion affecting the left pubic ramus	Left pubic ramus lesion
Vaidya et al. [14]	45	M	Paranasal sinus, nasal cavity, bilateral cervical nodes	MRI: sino-nasal masses, dural-based left frontal lesion	Dural-based left frontal lesion
Raslan et al. [15]	4	F	Right lacrimal gland	MRI: intensely enhanced bilateral lacrimal gland enlargement in T1-weighted	Left lacrimal gland
Mahajan et al. [16]	NA*	NA	Bones, and non-avid intracranial lesions		Intracranial lesions

*NA: not available

## Discussion

This single-center study reviewed the value of ^18^F-FDG PET/CT for the initial diagnosis and disease evaluation of RDD. We demonstrated that ^18^F-FDG PET/CT could identify lesions that were not visible on conventional examinations, assess treatment efficacy, and help adjust treatment regimens by distinguishing whether the lesions were active or not. We also reviewed the results of previous RDD cohort studies to reveal the higher sensitivity and lower false-positive rates of ^18^F-FDG PET/CT in comparison to conventional imaging techniques.

Most of the patients with RDD who had visited our center only had skin involvement. They typically visited the dermatology department without further imaging examination or treatment except for simple surgical resection and ultrasonography, which is consistent with the findings of Goyal et al. [18]. Cutaneous RDD [7] has a higher incidence in Asian and Caucasian patients. It rarely presents with systemic or extracutaneous involvement and is consequently classified as the C group of histiocytosis (cutaneous and mucocutaneous histiocytosis) [3], while the classical RDD with lymph node involvement belongs to the R group (Rosai–Dorfman disease and miscellaneous noncutaneous, non-Langerhans cell histiocytosis).

Many extranodal lesions at our center were located in the upper respiratory tract. Patients often presented with voice alterations or expiratory dyspnea [19]. Although surgical excision was indicated, these symptomatic airway diseases were often complicated by multisystem involvement and had some degree of postoperative relapse [19, 20]. While RDD is typically self-limiting [21], timely systemic therapy should be more effective than simple surgical resection in multifocal or refractory cases. Therefore, early whole-body assessment and follow-up by PET/CT is helpful for the timely identification of multifocal or progressive disease and for adjusting treatment regimens.

The general attitude toward the use of PET/CT in RDD was neutral [10]. At our center, ^18^F-FDG PET/CT was used to guide biopsy sites, recurrence assessment and pre-treatment whole body evaluation based on the clinical symptoms, especially in patients with extra-nodal manifestation or recurrent disease. When lesions found by CT or MRI were suspected of being malignant, PET/CT was often performed to help with differential diagnosis and selection of biopsy sites. Similar to the results of previous studies [16], our study supports the superiority of PET/CT for initial staging and treatment response assessment, especially in patients with disease relapse and progression. First, based on systemic evaluation, PET/CT was conducive to biopsy site selection and improved the positivity rate of subsequent diagnoses [22, 23] for RDD patients. As most RDD-involved sites are FDG-avid, PET/CT has advantages over traditional imaging for detecting occult sites in lymph nodes, bone, and residual lesions after surgical resection [24]. Additionally, although limited in sample size, several studies have investigated how PET/CT could be applied to follow-up therapeutic efficacy and optimize treatment regimens. Decreased FDG uptake indicates effective treatment or spontaneous recovery of RDD [25–30]. Despite the various treatment regimens of RDD, including glucocorticoids, chemotherapy, immunomodulators, and targeted therapy, no standardized treatment plan has been agreed upon. Therefore, timely and intuitive assessment of the treatment efficacy through ^18^F-FDG PET/CT is critical for determining effective options when selecting new treatments.

As different types of histiocytosis have different imaging distribution patterns, ^18^F-FDG PET/CT was considered a useful modality to differentiate assorted histiocytosis types [31], especially ECD. However, typical lesions of ECD, such as symmetric cortical osteosclerosis [32], retroperitoneal mass, and cardiac involvement [33], were occasionally observed in RDD [34–37], which led to confusing diagnoses. In our cohort, ECD was considered in three patients due to pituitary and bone lesions. However, skeletal system lesions in these cases were principally osteolytic rather than sclerotic, which indicated a different manifestation than ECD. Besides, because of the overlapping image pattern and association of different types of histiocytosis [38, 39], histopathological or even genetic testing, for example for BRAF mutations, is still recommended for distinguishing RDD from other histiocytosis. The most important function of PET/CT is to assist in obtaining sufficient biopsy tissue for establishing a rigorous diagnosis [10].

There were some non-FDG-avid lesions, which might indicate false negatives of this imaging approach, but only some of them underwent tissue biopsy and observation of therapeutic response to verify the etiology. Although a full-body PET/CT protocol is recommended to guarantee that lower extremity involvement is assessed, the rate of clinical implementation is relatively low. In addition to the scanning field, the possible causes for the low avidity of FDG might include comorbidities and treatment response. Nonetheless, in our study, the uptake value in patients with clinical manifestations of disease progression or exacerbation was not significantly influenced by medication. Hence, more prospective studies are needed to identify the proportion of false negatives and the corresponding histopathological features of PET/CT in patients with RDD.

Our study had several limitations. First, the retrospective nature and literature review of retrospective studies resulted in selection bias, and the patients incorporated in this cohort mainly had recurrence or extranodal involvement. The ability of ^18^F-FDG PET/CT to explore potential cutaneous RDD lesions should be further investigated. Second, histopathological identification of the involved sites could not all be obtained by biopsy or surgical excision, which might conceal some overlapping diseases and interference from relevant lesions, such as lymphoma and autoimmune diseases [10, 13, 40]. Although we thoroughly reviewed all medical records together with clinicians and few comorbidities were found, we cannot fully exclude the possibility. Additionally, the scanning filed usually only covered the skull vertex to mid-thigh level, which might lead to missing lower extremity involvement. Lastly, the sample size and number of continuous follow-ups for each patient using PET/CT were inadequate. Thus, larger prospective cohort studies are needed to analyze the correspondence between disease activity changes and ^18^F-FDG PET/CT results in patients with RDD.

## Conclusion

Generally, ^18^F-FDG PET/CT is beneficial to displaying the holistic characteristics of RDD and could compensate for some disadvantages of CT and MRI. Tissue biopsy of high metabolic sites suggested by PET/CT facilitates timely and accurate pathological diagnosis. Systemic assessment through PET/CT is also conducive to clinicians in monitoring disease activity as well as selecting and adjusting systemic therapeutic regimen.

## Data Availability

The datasets used and analysed during the current study are available from the corresponding author on reasonable request.

## Abbreviations

RDD: Rosai–Dorfman disease
FDG: Fluorodeoxyglucose
PET: Positron emission tomography
CT: Computed tomography
SUV: Standard uptake value
MRI: Magnetic resonance imaging
ECD: Erdheim–Chester disease
CNS: Central nervous system
